# The *nrfA*-type microbial communities are widespread in hot springs of the Tibet-Yunnan geothermal zone

**DOI:** 10.3389/fmicb.2025.1540611

**Published:** 2025-07-15

**Authors:** Xi Chen, Geng Wu, Zhicheng Yu, Fangming Li, Hongchen Jiang

**Affiliations:** State Key Laboratory of Biogeology and Environmental Geology, China University of Geosciences, Wuhan, China

**Keywords:** hot springs, Tibetan-Yunnan geothermal zone, *nrfA* gene, DNRA, microbial community

## Abstract

The microorganisms are main drivers of biogeochemical processes in geothermal ecosystems. The dissimilatory nitrate-to-ammonium reduction pathway (DNRA) could act as an alternative source of ammonium and provide an important nitrogen supply for the maintenance of geothermal ecosystems. Investigating the distribution of DNRA-functional bacteria is of great significance to understanding the source of biological nitrogen production in geothermal environments. In this study, we characterized the community distribution of microorganisms harboring *nrfA* genes in the sediments of hot springs from the Tibet-Yunnan geothermal zone, with the use of Illumina MiSeq high-throughput sequencing of *nrfA* genes and R language software for statistical analysis. In the present study, the *nrfA* genes were successfully amplified from the hot springs with a temperature of 38°C–80°C. The *nrfA*-based phylogenetic analysis showed that the DNRA pathway is widespread within the geothermal ecosystems, with microorganisms harboring *nrfA* genes predominantly belonging to phyla *Chloroflexi*, *Proteobacteria*, *Deinococcus-Thermus* (top 10), etc. Genus-level analysis revealed *Thermoflexus (Chloroflexi)* as the dominant taxon in the DGQ, while *Geothrix (Acidobacteria)* showed peak abundance in weakly acidic sites. The DNRA-functional community structure and *nrfA* gene abundance also showed a sample variability, even among samples from the same region, there were differences in dominant populations and overall *nrfA* gene abundance between them. Statistical analysis results indicate that the distribution of *nrfA* type microorganisms was mainly influenced by physicochemical factors, including pH, SO_4_^2−^, and NO_2_^−^ concentrations. These findings deepen our understanding of the nitrogen cycle in extreme environments and provide valuable perspectives on the role of nitrogen metabolism in both contemporary and ancient geothermal systems.

## Introduction

Terrestrial hot springs represent natural laboratories for studying extremophile ecology, characterized by their dual extremes of elevated temperatures and oxygen-depleted conditions. In these ecosystems, microorganisms are the main drivers of biogeochemical processes, including the nitrogen cycle, which is essential for ecosystem stability. Nitrogen is one of the most vital elements and an important nutrient that often limits biological productivity on Earth (Kuypers et al., 2018). In hot springs, microorganisms play a crucial role in converting various forms of nitrogen and are therefore central to the nitrogen cycle in this environment (Gruber and Galloway, 2008). Previous investigations of the Tibet-Yunnan geothermal zone have revealed diverse microbial communities dominated by thermophilic *Chloroflexi*, *Aquificae*, and methanogenic archaea (Song et al., 2013). However, the functional potential of DNRA microorganisms in these ecosystems remains uncharacterized. Nitrogen availability remains a limiting factor for the stability and sustainability of geothermal ecosystems under extreme environmental conditions. One nitrogen-containing compound of particular interest in hot springs is ammonium. It serves as an important source of nitrogen in geothermal habitats. Ammonium can act as an electron donor for ammonia-oxidizing bacteria or can be taken up by other microorganisms as a nitrogen source (Bernard et al., 2015; Burgin and Hamilton, 2007; Su et al., 2020; Zhang Y. et al., 2023). Ammonium dynamics in hot springs, driven by microbial production and consumption processes, play a pivotal role in sustaining ecosystem functionality through dual mechanisms: fueling microbial biomass and maintaining nitrogen flux under extreme conditions.

Nitrogen availability is a key limiting factor for the stability of hot spring ecosystems. Microorganisms often need to convert natural nitrogen sources into ammonium before they can use it (Canfield et al., 2010). This conversion occurs primarily through nitrogen fixation and the dissimilatory reduction of nitrate to ammonium. Several different microbial nitrogen cycling processes are active in hot springs, including nitrogen fixation, nitrification, ammonification, denitrification, anaerobic ammonia oxidation and dissimilatory nitrate reduction to ammonium (DNRA). Terrestrial hot springs are characterized by low oxygen levels, classified as oceanic oxygen minimum zones (OMZs) on Earth, and host unique microbial communities that rely on alternative electron acceptors for respiration in the absence of oxygen (Lam and Kuypers, 2011). Following the electrochemical sequence, nitrate (NO_3_^−^) is the preferred electron acceptor after oxygen (Canfield et al., 2005). However, in ecosystems where denitrification dominates, nitrate reduction leads to nitrogen loss. Therefore, the recycling of nitrate through pathways like DNRA becomes critical for sustaining microbial growth in these nitrogen-limited environments. DNRA is a key anaerobic pathway for ammonium production, in which microorganisms reduce nitrate or nitrite to ammonium while simultaneously breaking down organic matter without causing nitrogen losses (Cao et al., 2016; Kaviraj et al., 2024). In low-oxygen, high-organic-carbon, or reducing environments, such as sediments, wetlands, and hot springs, DNRA serves as a critical source of bioavailable ammonium due to its nitrogen retention function. Compared to other nitrogen forms, ammonium can be directly assimilated by microorganisms, supporting their growth more efficiently. While both nitrogen fixation and DNRA contribute to ammonium supply in hot springs, the extreme temperatures often limit the survival of higher organisms, nitrogen-fixing enzymes are found predominantly in photosynthetic microorganisms such as unicellular cyanobacteria (Seckbach and Oren 2007). However, photosynthetic activity is somewhat inhibited under high-temperature conditions (Cox et al., 2011), making the DNRA pathway an indispensable source of ammonium in these extreme environments. This suppression of nitrogen fixation positions DNRA as a potentially dominant pathway for ammonium generation in geothermal systems, particularly under thermophilic conditions. Studying DNRA in hot springs is essential for unraveling microbial-driven nitrogen cycling and survival mechanisms in extreme habitats, further highlighting its ecological significance.

Recent studies have highlighted the importance of the DNRA pathway in aquatic ecosystems. For example, Giblin et al. (2013) found that DNRA was responsible for 30% of nitrate reduction in 26 of 55 coastal areas. Similarly, Song et al. (2014) found that the DNRA pathway was responsible for 44–75% of nitrate reduction in the Cohen Estuary, with higher organic matter content corresponding to increased DNRA bacterial activity and increased *nrfA* gene abundance (Song et al., 2014). In freshwater ecosystems, organic carbon (OC) and nitrate (NO_3_^−^) concentrations have been identified as key factors regulating the efficiency of denitrification and DNRA. Numerous field studies suggest that DNRA outperforms denitrification in environments with high carbon-nitrogen ratios (Burgin and Hamilton, 2007; van den Berg et al., 2016). Furthermore, DNRA is closely linked to the sulfur cycle; for example, high concentrations of sulfide have been shown to increase DNRA rates because sulfide serves as an efficient electron donor for DNRA bacteria (Zhu et al., 2018). Other environmental factors, such as organic matter content, nitrogen sources, water temperature, and dissolved oxygen also influence the pathway of nitrate reduction pathway in different environments (Bu et al., 2017; Washbourne et al., 2011). While denitrification and anammox are recognized as dominant nitrogen loss pathways in many ecosystems, their relative contributions in high-temperature environments remain unclear. In contrast to nitrogen-removal processes, DNRA retains nitrogen within the system through ammonium production—a feature particularly advantageous in nitrogen-limited hot springs. Quantitative assessments of DNRA-driven nitrogen flux in geothermal ecosystems remain scarce. While denitrification has dominated previous nitrogen cycling studies in hot springs, emerging evidence suggests DNRA may contribute substantially to nitrogen retention. In the Great Basin hot springs (USA), DNRA accounted for up to 40% of total nitrate reduction in high-temperature sediments (60–75°C), with functional genes (*napA, nirB, nrfA*) predominantly associated with thermophilic fermentative bacteria and archaea (Dodsworth et al., 2011; Hedlund et al., 2011). However, enzymatic thermal thresholds constrain this process, as DNRA activity was suppressed above 84.4°C in some systems (Everroad et al., 2012). Notably, extreme thermophiles like *Pyrolobus fumarii* demonstrate DNRA capability even at 113°C (Hafenbradl et al., 1997), suggesting unrecognized thermo-adaptive mechanisms in high-temperature niches. These findings highlight both the potential significance and temperature-dependent variability of DNRA contributions in geothermal nitrogen budgets.

This limited knowledge regarding microbial nitrogen transformation mechanisms creates a barrier to advancing our understanding of biogeochemical cycling in extreme environments. Therefore, investigating the prevalence of DNRA processes in hot springs, as well as elucidating the structure of the microbial communities involved and the responses to environmental factors, is of significant value. This study investigates the community structure of dissimilatory nitrate reduction to ammonium (DNRA) microorganisms in Tibetan-Yunnan hot spring sediments and their interactions with environmental factors. Focusing on high-temperature geothermal ecosystems, it addresses the limited domestic research on DNRA microbial ecology by elucidating the composition, distribution patterns, and environmental drivers of these functional communities, thereby providing foundational insights into nitrogen cycling processes in the Tibet-Yunnan geothermal zone. Understanding the characteristics of the potential DNRA functional groups in hot springs and their factors influencing their activity can lead to a deeper understanding of the nitrogen cycling mechanisms in extreme environments. These findings not only improve our understanding of nitrogen cycling in contemporary geothermal systems (thermally active springs with ongoing geological activity), but also provide a framework for exploring nitrogen cycling activities on the early Earth.

## Materials and methods

### Description of the research area

The Tibet-Yunnan geothermal zone, located in southwestern China, encompasses the Tibet geothermal area and the Tengchong geothermal region in Yunnan. In the Tibetan geothermal area, tectonic activity is influenced by the subduction of the Indian lithosphere, the thickening of the Tibetan crust, and the eastward extrusion of Tibetan lithospheric material. In addition, rapid easterly flow of deep crustal material has formed a series of east–west trending tectonic features along the Himalayan foothills and the Indus-Yarlung-Tsangpo suture belt. These structural features have resulted in numerous intermittent geothermal vents throughout the region (Royden et al., 2008). The Tengchong geothermal region of Yunnan, located at the junction of the Eurasian and Indian plates, was shaped by recent volcanic activity and magmatic intrusions from the Tibetan Plateau. These processes have resulted in active geothermal phenomena, including boiling springs, ejecta springs, intermittent geysers, and hydrothermal explosions. Neotectonics movements since the Quaternary have also contributed to the migration of magmatic activity toward the tropical marine area. This has made the Tengchong Rehai region a hotspot for magmatic eruptions. The geological framework of the Rehai area is also characterized by several parallel and reverse faults and granite conglomerate blankets. These features create favorable conditions for the formation of geothermal geysers and contribute to the region’s rich geothermal activity.

### Samples collection

**Figure 1:**
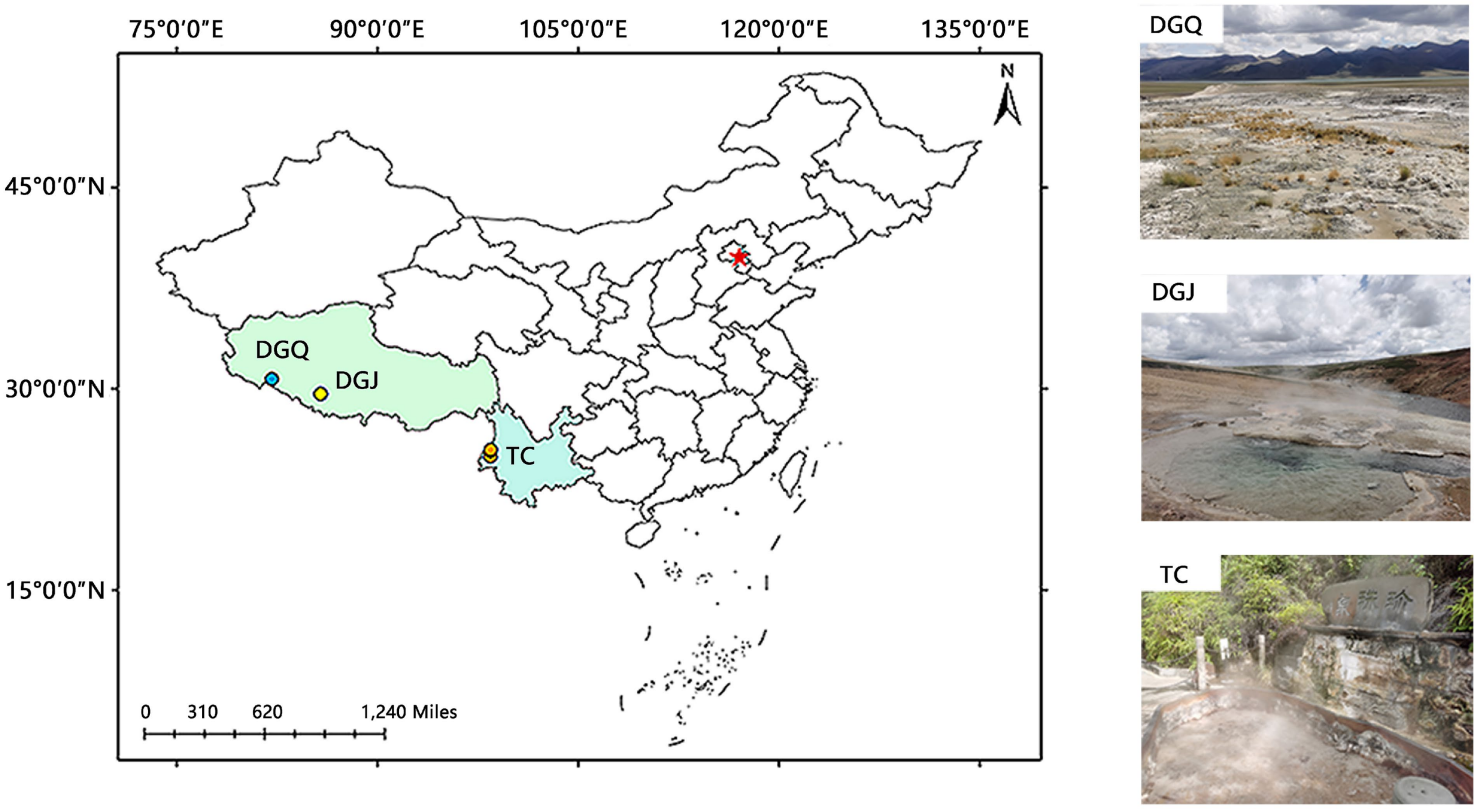
Map depicting the locations of the studied area within the Tibet-Yunnan geothermal zone. TC, Tengchong, Yunnan; DGJ, Dagejia, Tibet; and DGQ, Duoguoqu, Tibet.

In this study, a total of 53 hot spring samples were collected from three regions: Tengchong area (TC) in Yunnan, Dagejia (DGJ) and Duoguoqu (DGQ) areas in Tibet (Figure 1).

Sampling included bottom water (deep water near the sediment layer) and surface sediment (0–10 cm) from the hot springs. At each site, three replicate samples were collected for molecular experiments and an additional three samples were collected to determine physicochemical properties. Sediment samples were collected with sterilized spatulas, then placed in sterile centrifuge tubes, and transported to the laboratory on dry ice. A portion of the sediment samples was provided for physicochemical analysis, while the remaining samples were stored at −80°C for subsequent DNA extraction and molecular experiments. Hot spring water samples were collected in 50 mL bottles, filtered through 0.22 μm cellulose acetate membranes, and stored on dry ice. These water samples were later analyzed for anions, cations and other elemental compositions (Supplementary Section 2).

### Physicochemical analyses

The geographic coordinates of the sample sites were recorded using a portable GPS device (eTrex H, United States). The pH and temperature of the hot springs were measured *in situ* using a portable pH meter SX711 (Shanghai Sanxin Instrument Factory, China). The water chemistry parameters, including Fe^2+^ and NO₂^−^, were determined spectrophotometrically using a Hach kit (Hach, United States). Hot spring water samples (25–35 mL) were filtered through 0.22 μm nitrocellulose or polycarbonate membranes, acidified with concentrated HNO₃ to a final concentration of 0.1 M, and transported to the laboratory for further analysis. Major anions and cations, such as Br^−^, NO₃^−^, SO₄^2−^, Mg^2+^, and Ca^2+^, were quantified using a Dionex DX-600 ion chromatograph (Thermo Fisher Scientific Inc., United States). Sediment samples were processed for nutrient analysis using potassium persulfate digestion. Total nitrogen (TN) in the sieved sediment was measured with a UV spectrophotometer after freeze-drying and grinding. The total organic carbon (TOC) in the sediment was determined using an elemental analyzer, and the carbon-to-nitrogen (C/N) ratio was calculated (Supplementary Section 2) (Ma et al., 2021).

### DNA extraction and quantitative real-time PCR (q-PCR) analysis

DNA was extracted from the 53 sediment samples using the FastDNA SPIN Kit for soil (MP Biomedicals, LLC, Solon, OH), processing 0.5 g of sediment per sample. The extracted DNA was subsequently amplified and purified by PCR to isolate the DNRA microbial community. The *nrfA* functional gene, associated with the dissimilatory nitrate reduction to ammonium (DNRA) pathway, was specifically targeted for amplification and purification using primers *nrfA*-F2awMOD (5′ – GSICARTGYCAYGTIGARTA – 3′) and *nrfA*-R1MOD (5′ – GGCATRTGRCARTCIRYRCA – 3′). The *nrfA* primers (*nrfA*-F2awMOD/*nrfA*-R1MOD) were validated *in silico* and experimentally by Cannon et al. (2019), showing specificity to *nrfA* genes (Supplementary Figure S1) (Cannon et al., 2019). In this study, PCR products from 23 samples were confirmed via agarose gel electrophoresis (Supplementary Figure S2), and amplicon sizes matched the expected fragment length (~270 bp). and the positive amplification of *nrfA* genes was confirmed via 1% agarose gel electrophoresis The reaction mixture contained the following components: 2.5 μL of 10 × PCR buffer, 2 μL of deoxyribonucleoside triphosphates (dNTPs, 2.5 μM, Sangon Biotech, Shanghai), 0.3 μL of Takara Ex Taq DNA polymerase (5 U/μl, Takara Bio, Japan), 1 μL each of forward and reverse primers (10 μM), 0.5 μL of bovine serum albumin (BSA, Takara Bio, Japan), 1 μL of sample DNA, and sterile ddH₂O to a final volume of 25 μL. The PCR conditions were as follows: pre-denaturation at 95°C for 5 min, 40 cycles of denaturation at 95°C for 30 s, annealing at 56°C for 30 s and extension at 72°C for 30 s, followed by a final extension at 72°C for 10 min. To verify amplification, 5 μL of the PCR product was analyzed using 1% agarose gel electrophoresis. Each PCR reaction was performed in triplicate to minimize experimental errors, and three technical replicates were conducted for each DNA sample. The PCR amplifications were performed using a Bio-Rad T100 thermocycler (Bio-Rad Laboratories, United States). A blank control, containing sterile deionized water instead of DNA, was included in the same reaction system to exclude reagent contamination. Positive PCR products were purified using an agarose gel electrophoresis method. Gel sections containing the target DNA bands were excised, and DNA was extracted and purified using the Axygen purification kit (Axygen, Union City, CA, United States) with the centrifugation protocol. DNA concentrations were quantified using a NanoDrop ND-1000 spectrophotometer (NanoDrop Technology, DE, United States).

The abundance of the *nrfA* gene was quantified using quantitative real-time fluorescence PCR (qPCR) on a CFX Connect instrument (Bio-Rad Laboratories, Inc.) using the SYBR Green II method. The qPCR reaction was performed in a 20 μL system: 10 μL 2 × SYBR Premix Ex Taq enzyme (Takara Bio, Japan), 1 μL each of forward and reverse primers (10 μM), 1 μL sample DNA, and ddH2O supplemented to a total volume of 20 μL. Amplification and sequencing were performed as follows: pre-denaturation at 95°C for 5 min, 40 cycles of denaturation at 95°C for 30 s (based on optimized experimentally), annealing at 56°C for 30 s and extension at 72°C for 30 s. Amplification included a melting curve analysis (65°C to 95°C, increments of 0.5°C per 5 s) to confirm specificity. Fluorescence signals were acquired during the extension step (72°C). Plasmid standards with known concentrations and copy numbers were used to generate a standard curve for quantifying the abundance of DNRA bacteria. Eight serial 10-fold dilutions of the plasmid were prepared, with each gradient analyzed in triplicate. Negative controls, using ddH₂O as template, were provided in triplicate for each amplification run. All sample reactions were also conducted in triplicate to ensure accuracy. The standard curve, constructed using the plasmid standards, allowed the determination of *nrfA* gene abundance in the samples.

### High-throughput sequencing of *nrfA*

The 23 samples with successful PCR amplification results were selected for high-throughput sequencing, and amplification was performed using *nrfA* functional primers with barcode tags. The sequence used for amplification was identical to that used for PCR sequencing, but barcode-tagged primers incorporated 12-bp barcode sequences into the forward primers and sequencing adapters to differentiate the samples. The forward primers contained Illumina adapter sequences (5’-TCGTCGGCAGCGTCAGATGTGTATAAGAGACAG-3′) and 12-bp barcodes, while reverse primers included the reverse adapter (5’-GTCTCGTGGGCTCGGAGATGTGTATAAGAGACAG-3′). The amplification system, conditions, and purification method followed the same protocol as the initial PCR amplification. For the subsequent high-throughput sequencing, the concentration of the purified sample DNA had to exceed 30 ng/μl.

High-throughput sequencing was conducted using the Illumina MiSeq platform (2 × 300 bp) (Guangdong MAGIGENE Technology Co., Ltd.). Sequencing depth per sample (Supplementary Table S1) averaged 60,000 reads. The sequencing data were categorized according to the OTU principle, and the high throughput sequences were quality checked and screened according to the previous experimental methods to ensure the reliability of the OTU classification to improve the accuracy of the annotation of the DNRA functional groups of the samples and to make the conclusions of the statistical analysis of the study more scientific. The specific processing method is as follows: the obtained paired-end raw sequences were quality filtered by Trimmomatic. The pre- and post-sequences obtained by amplification were spliced using the default settings of FLASH (Magoč and Salzberg, 2011), and the sequences were assigned to each sample based on their unique barcode, and then the barcodes and primers were removed. Valid sequences were trimmed at 97% similarity to group them into an operational taxonomic unit (OTUs) using USEARCH software (Edgar et al., 2011), and chimeric sequences and sequences of individual OTUs were excluded to avoid possible bias. Classification annotation information was performed for the representative OTUs sequences obtained using the SILVA database^1^ and the non-redundant protein sequence database^2^, the OTU tables were normalized and single instances were removed from each sample. The resulting standard OTU tables were then used for subsequent analyses.

### Statistical analysis

This study employed R language software (version 4.1.0) and Chiplot platform^3^ for statistical analysis. R software was used for alpha diversity and correlation tests, and the Chiplot platform was applied for Mantel tests and RDA. Alpha diversity analysis was conducted to assess biodiversity in the samples containing the *nrfA* gene, focus on species richness, diversity and evenness, and the statistical tests for alpha diversity (Supplementary Table S2) included ANOVA and Kruskal–Wallis. Relative abundance histograms were constructed in R to compare community abundance between samples. Pearson correlation analysis was applied to assess the relationships between the physicochemical properties of sampling sites and microbial communities. Mantel tests were performed via the Chiplot platform and included OTUs, *nrfA* functional gene abundance, and physicochemical data. Principal co-ordinate analysis (PCoA) and principal component analysis (PCA) were conducted to perform unconstrained ordination of community structure and environmental properties of each sample sites. To explore the effects of physicochemical properties and spatial factors on microbial community structure and composition (including microorganisms containing *nrfA* functional genes), redundancy analysis (RDA) was performed. Environmental factors autocorrelation was tested using the “Hmisc” package in R. To identify multicollinearity among explanatory variables, Variance Inflation Factors (VIFs) were calculated (Blanchet et al., 2008), when 0 < VIF < 10, there is no multicollinearity; when 10 ≤ VIF < 100, there is strong multicollinearity. When VIF > 10 (i.e., there is obvious covariance between the variables and other environmental variables), it is necessary to remove them from subsequent analyses until VIF < 10 among the variables. The Principal Coordinates of Neighborhood Matrix (PCNM) method was used to decompose geographic coordinates of sampling sites into spatial variables. This method decomposes geographic coordinates into spatial variables to quantify the influence of spatial distance on microbial community structure. Significant spatial variables (*p* < 0.05) were selected for Variance Partitioning Canonical Correspondence Analysis (VPA) to quantify the relative contributions of environmental and spatial variables to bacterial community structure. The proportion of unexplained variance was attributed to factors not measured in the study.

## Results

### Physicochemical parameters and screening of positive samples

**Figure 2:**
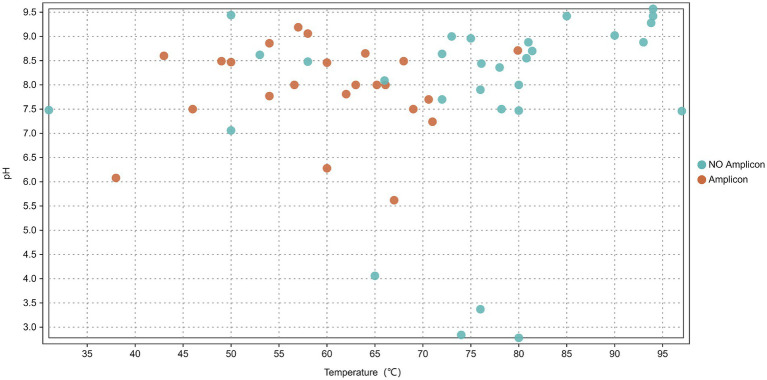
Distribution of *nrfA* gene across 53 sediment samples in relation to spring water pH and temperature. Orange indicates environments where amplicons were detected while blue represents environments with no amplicons detected.

In this study, the *nrfA* gene was successfully amplified from 23 out of 53 hot spring sediment samples (based on successful PCR amplification) (Figure 2), with no amplification in negative controls (Supplementary Figure S2), including 15 samples from Tibet (DGJ2, DGJ4, DGJ5, DGJ8, DGJ9, DGJ11, DGJ13, DGJ14, DGQ1, DGQ2, DGQ4, DGQ5, DGQ6, DGQ7, DGQ9) and 8 samples from Tengchong, Yunnan (HMZ3, JXH, JXL, JM, JZ, ZY, WGQ, QQ) (Table 1).

**Table 1:** Geographic coordinates and physicochemical parameters of 23 positive hot springs in the study area.

Sample	Sites	GPS(N)	GPS(E)	Altitude (m)	pH	Temp (°C)	Fe^2+^ (mg/L)	NO_2_^−^(mg/L)	SO_4_^2−^(mg/L)	Br^−^ (mg/L)	NO_3_^−^(mg/L)	C/N
DGJ2	DGJ	29.59887	−85.75072	5,051	8.86	54	0.00	13.39	88.25	1.80	1.05	13.38
DGJ4	DGJ	29.59995	−85.74995	5,068	5.62	67	3.90	13.79	93.24	1.81	1.27	126.97
DGJ5	DGJ	29.60018	−85.75017	5,075	6.28	60	1.35	1.13	168.19	1.95	3.57	0.09
DGJ8	DGJ	29.60084	−85.74952	5,073	9.06	58	0.00	12.30	91.37	1.80	1.00	3.37
DGJ9	DGJ	29.60089	−85.74902	5,070	8.49	68	0.05	18.19	99.95	2.44	2.93	6.99
DGJ11	DGJ	29.60092	−85.74902	5,070	7.77	54	0.07	13.90	73.52	1.46	1.78	0.52
DGJ13	DGJ	29.60176	−85.74923	5,070	8.49	49	0.00	14.10	74.65	1.45	1.81	5.19
DGJ14	DGJ	29.60287	−85.75122	5,073	8.71	79.9	0.01	12.20	76.83	1.43	3.20	0.08
DGQ1	DGQ	30.70525	−82.10560	4,861	7.5	69	0.00	18.61	66.96	2.67	1.16	0.23
DGQ2	DGQ	30.70527	−82.10550	4,862	7.7	70.6	0.00	19.16	71.90	2.86	1.05	0.10
DGQ4	DGQ	30.70524	−82.10550	4,863	8	66.1	0.00	29.69	74.63	2.77	1.34	7.02
DGQ5	DGQ	30.70524	−82.10550	4,864	7.5	46	0.00	19.58	73.21	2.89	1.18	5.56
DGQ6	DGQ	30.70524	−82.10550	4,865	8	63	0.00	21.35	67.35	2.83	1.11	1.98
DGQ7	DGQ	30.70524	−82.10550	4,866	8	65.2	0.00	19.90	67.80	2.82	1.02	1.48
DGQ9	DGQ	30.70524	−82.10550	4,867	8	56.6	0.00	26.64	70.67	2.89	1.04	2.57
HMZ3	TC	24.95	−98.43805	1,350	9.19	57	0.04	13.02	45.07	3.20	1.84	18.65
JXH	TC	25.44119	−98.46016	1,682	8.65	64	0.02	19.82	26.80	0.00	0.00	4.26
JXL	TC	25.44119	−98.46016	1,682	8.6	43	0.00	1.96	29.57	2.74	1.74	10.52
JM	TC	25.44152	−98.45986	1,679	8.47	50	0.00	20.39	27.65	0.00	0.00	22.69
JY	TC	25.43944	−98.46083	1720	7.81	62	0.04	21.26	27.87	0.00	0.00	1.08
ZY	TC	25.43930	−98.45997	1,676	8.46	60	0.00	16.12	35.00	0.00	1.76	5.46
WGQ	TC	25.59472	−98.73888	1,425	6.08	38	1.17	1.09	149.27	0.00	0.00	3.73
QQ	TC	24.95055	−98.43777	1,337	7.24	71	0.42	6.40	113.35	2.92	1.69	0.54

The positive sampling sites had a temperature range of 38–80°C, with an average temperature of 59.6°C. Three samples (DGJ14, DGQ2 and QQ) were collected from sites with temperature above 70°C, 10 samples were collected from sites with temperatures between 60°C and 70°C, and five samples were collected from sites with temperatures below 50°C. The pH at the positive sample site ranged from 5.5 to 9.4, with most sites having neutral to alkaline conditions. The result suggests that the *nrfA* gene is widely distributed across various hot springs in the Tibet-Yunnan geothermal region, and the DNRA pathway is potentially widespread in these ecosystems.

### Quantification of *nrfA* gene abundance

**Figure 3:**
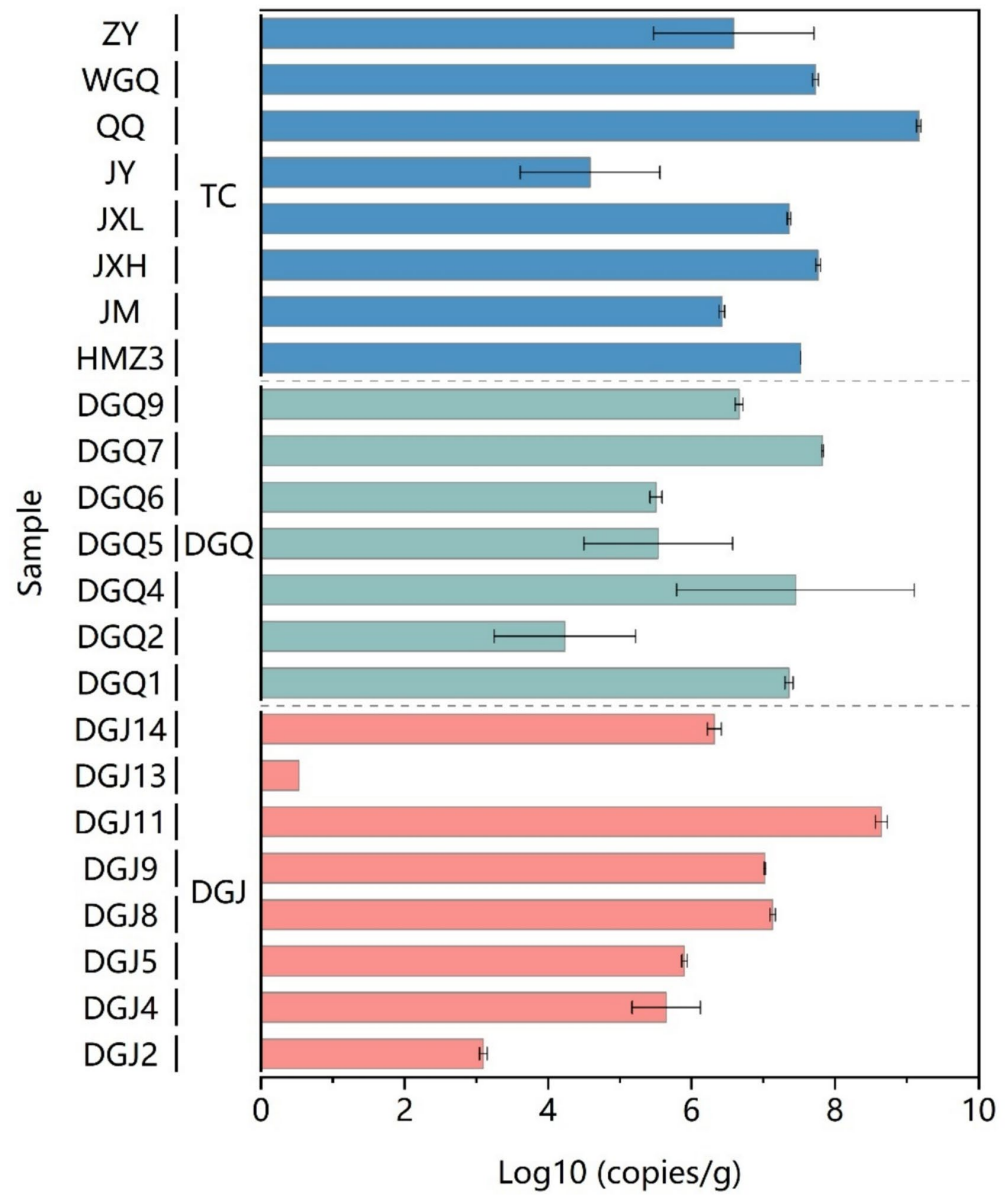
DNRA community abundance based on *nrfA* functional gene analysis in sediment samples from the study area.

The distribution of *nrfA* gene abundance varied significantly in the three regions. The highest *nrfA* abundance was recorded in the QQ sample from Tengchong, with a value of 1.45 × 10^9^ copies/g. On average, the *nrfA* gene abundance was significantly higher in the Tengchong region compared to the DGJ and DGQ regions. The lowest average *nrfA* abundance was observed in DGJ with a value of 3.43 × 10^5^ copies/g (Figure 3). Despite notable spatial and temporal differences in the distribution of *nrfA* genes in the three regions, the overall abundance remained consistently high. These results highlight the widespread occurrence and high abundance of DNRA microorganisms in the Tibet-Yunnan hot springs and highlight the importance of DNRA processes in the nitrogen cycling of these geothermal ecosystems.

### Diversity of functional microbial communities in DNRA

**Figure 4:**
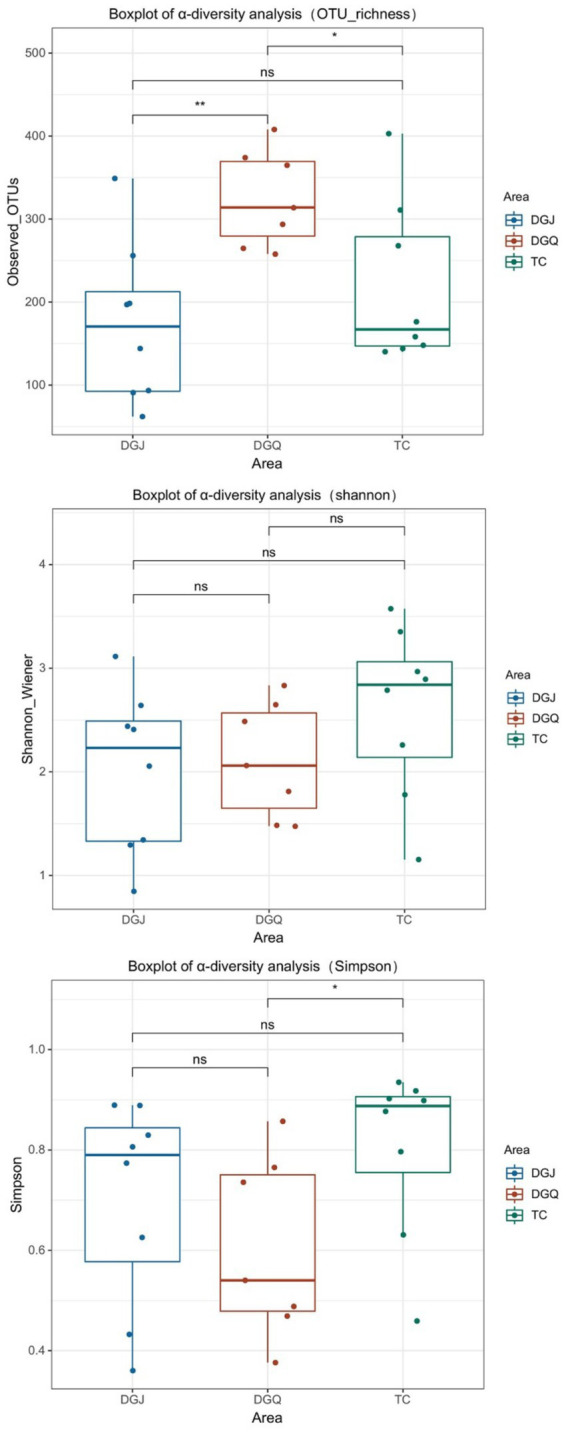
Box plots depicting the alpha diversity analysis of DNRA communities. Significance was determined by ANOVA and Kruskal Wallis test, followed by Bartlett’s test, Dunn test and Bonferroni correction. Statistical significance is marked as follows: *p ≤ 0.05, **p ≤ 0.01, ***p ≤ 0.001 and ****p ≤ 0.0001, and ns for no significant difference.

High-throughput sequencing of the *nrfA* gene in 23 positive hot spring samples yielded a total of 429,0310 high-quality sequences, classified into 6,991 operational taxonomic units (OTUs). Sequencing depth averaged 60,000 reads per sample (Supplementary Table S1), with rarefaction curves plateauing (Supplementary Figure S3). Alpha diversity metrics (Shannon, Simpson, observed OTUs) are summarized in Supplementary Table S2. To assess the *α*-diversity of the DNRA bacterial community, the Observed OTUs index was used to characterize community richness, while the Shannon and Simpson indices reflected diversity and evenness (Figure 4). The results showed that DNRA microbial richness was highest in the DGQ region, with similar OTU counts in the TC and DGQ regions. However, the Shannon and Simpson indices showed greater microbial diversity in the TC region, while the DGQ region had the lowest diversity. In the analysis of the Simpson index and Observed OTUs (richness), significant differences were observed between DGQ and TC (*p* < 0.05), while DGJ and DGQ exhibited highly significant differences in OTU richness (*p* < 0.01). However, no statistically significant differences were detected among the groups (DGQ, DGJ, TC) based on the Shannon-Wiener index. For statistical methodology, the Shannon-Wiener index and OTU richness data followed a normal distribution; thus, ANOVA with Bartlett’s test for homogeneity of variance was applied. In contrast, the Simpson index, due to its non-normal distribution, was analyzed using the Kruskal–Wallis non-parametric test, followed by *post hoc* Dunn’s test with Bonferroni correction for robust multiple comparisons.

**Figure 5:**
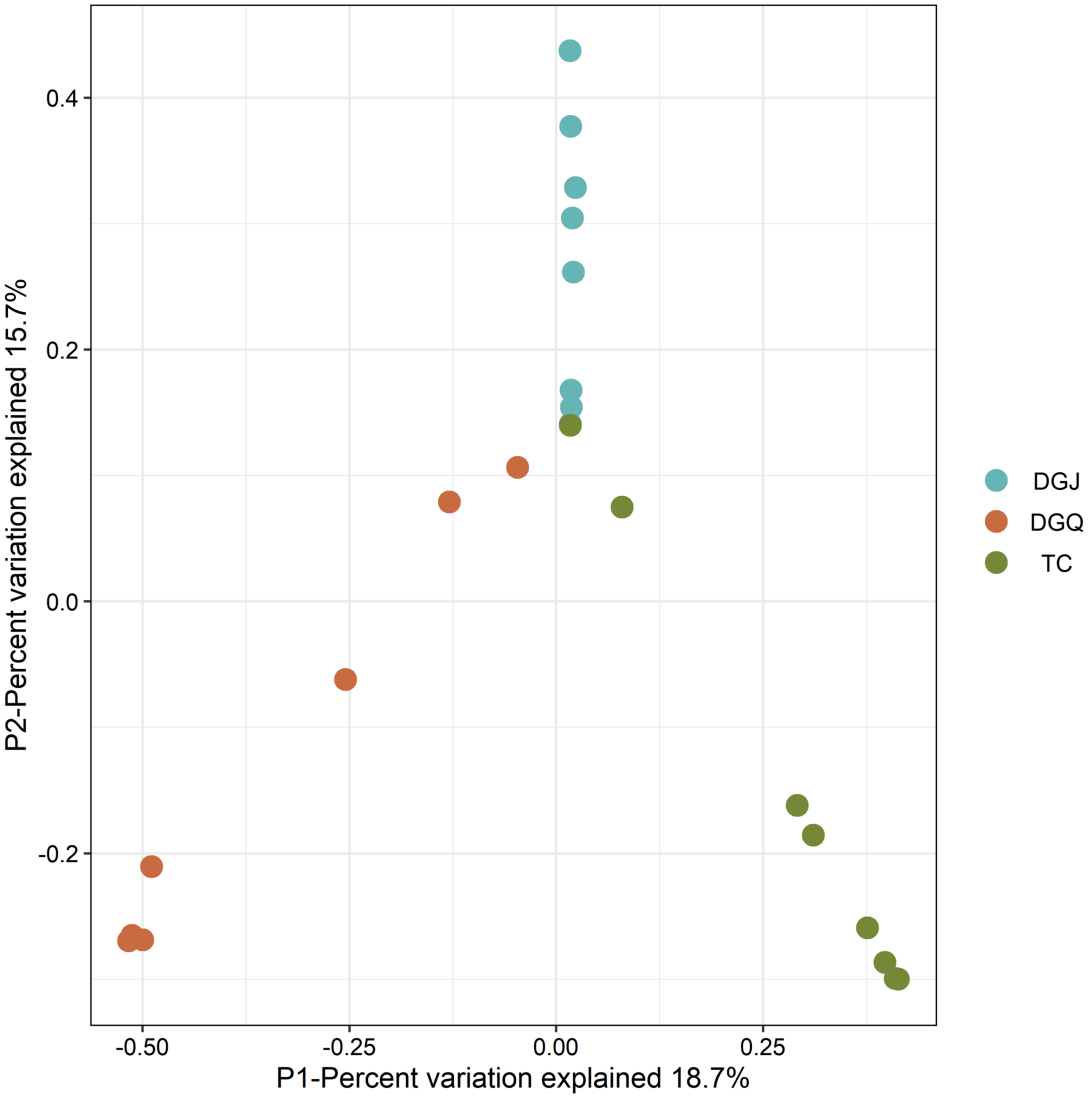
Principal coordinates analysis (PCoA) ordination of DNRA communities from positive sample sites. Each dot represents an independent biological sample. Proximity of points represents similarity in community structure, with horizontal and vertical axes represent different dimensions of the variable.

**Figure 6:**
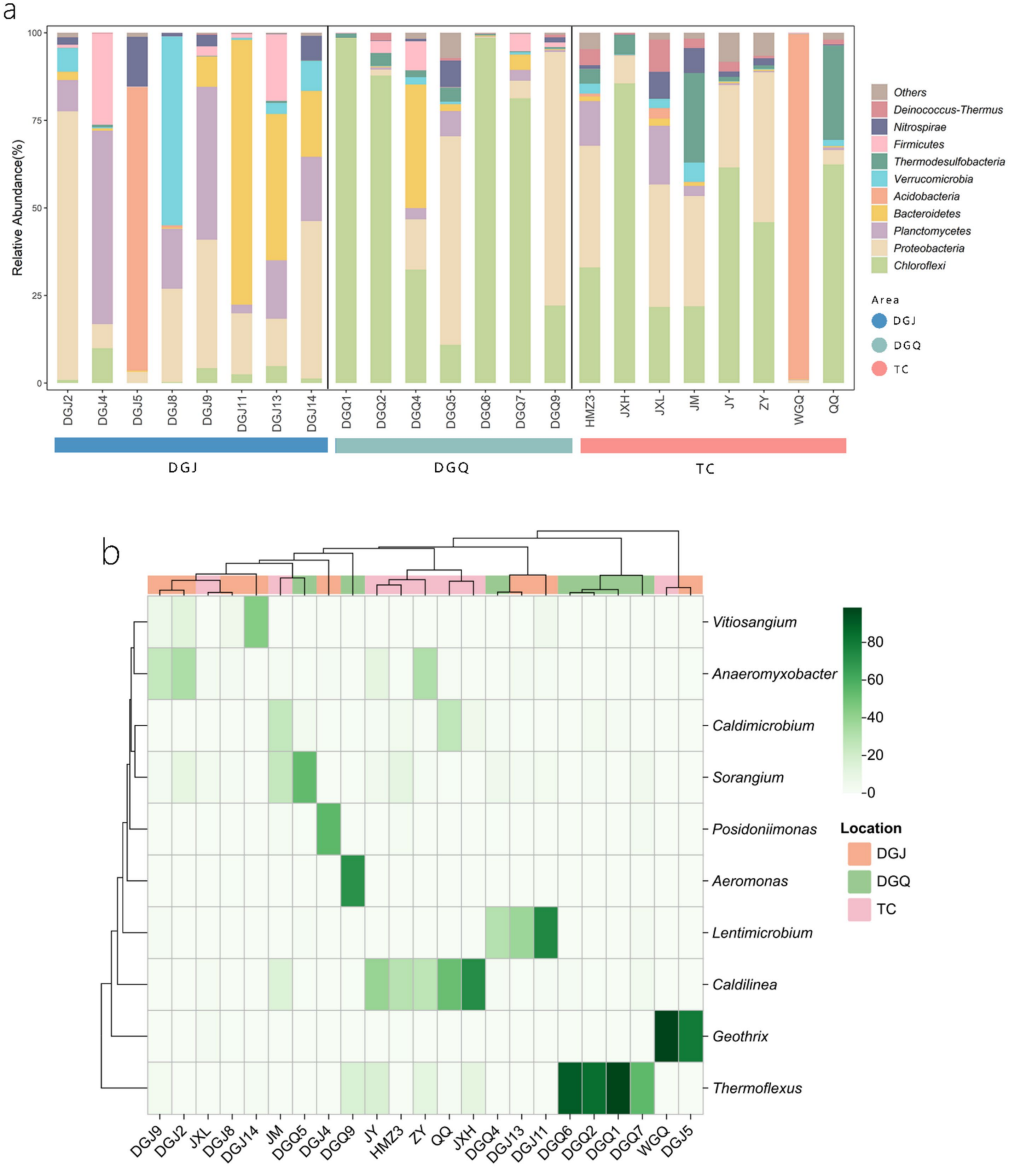
Phylogenetic analysis of *nrfA* gene sequences from positive hot spring samples **(a)**, heatmap showing DNRA bacterial composition at the genus level for positive hot spring samples across the study area **(b)**, color gradient indicates relative abundance (%) of genera across samples.

Principal coordinate analysis (PCoA) was performed to assess differences in phylum-level microbial community structure among the three regions (Figure 5). PCoA revealed significant structural divergence among most samples, with partial overlap only in a few. Each dot represents an independent biological sample. At the phylum level, a *nrfA*-based phylogenetic analysis identified the 10 microbial phyla with the highest relative abundance: *Chloroflexi* 34.23%, *Proteobacteria* 24.31%, *Planctomycetes* 9.2%, *Bacteroidetes* 8.55%, *Acidobacteria* 8.02%, *Verrucomicrobia* 3.92%, *Thermodesulfobacteria* 3.39%, *Firmicutes* 2.99%, *Nitrospirae* 2.49% and *Deinococcus-Thermus* 1.14% (Figure 6a). The dominance of *nrfA* genes in *Chloroflexi* and *Proteobacteria* was observed in almost all TC and DGQ samples and covered most of the hot spring sites. In contrast, the DGJ region exhibited a distinct community structure, with *Proteobacteria* being more prominent. To resolve finer taxonomic patterns, a heatmap of genus level OTU distributions (Figure 6b) revealed significant spatial heterogeneity in dominant DNRA taxa. For instance, *Thermoflexus* (*Chloroflexi*) dominated the Duoguoqu region (DGQ), while *Geothrix* (*Acidobacteria*) showed peak abundance in weakly acidic sites (DGJ5, WGQ). Other prevalent genera included *Caldilinea* (*Chloroflexi*) and *Lentimicrobium* (*Bacteroidetes*), whose clustering patterns suggested conserved functional guilds across springs despite geographic separation.

**Figure 7:**
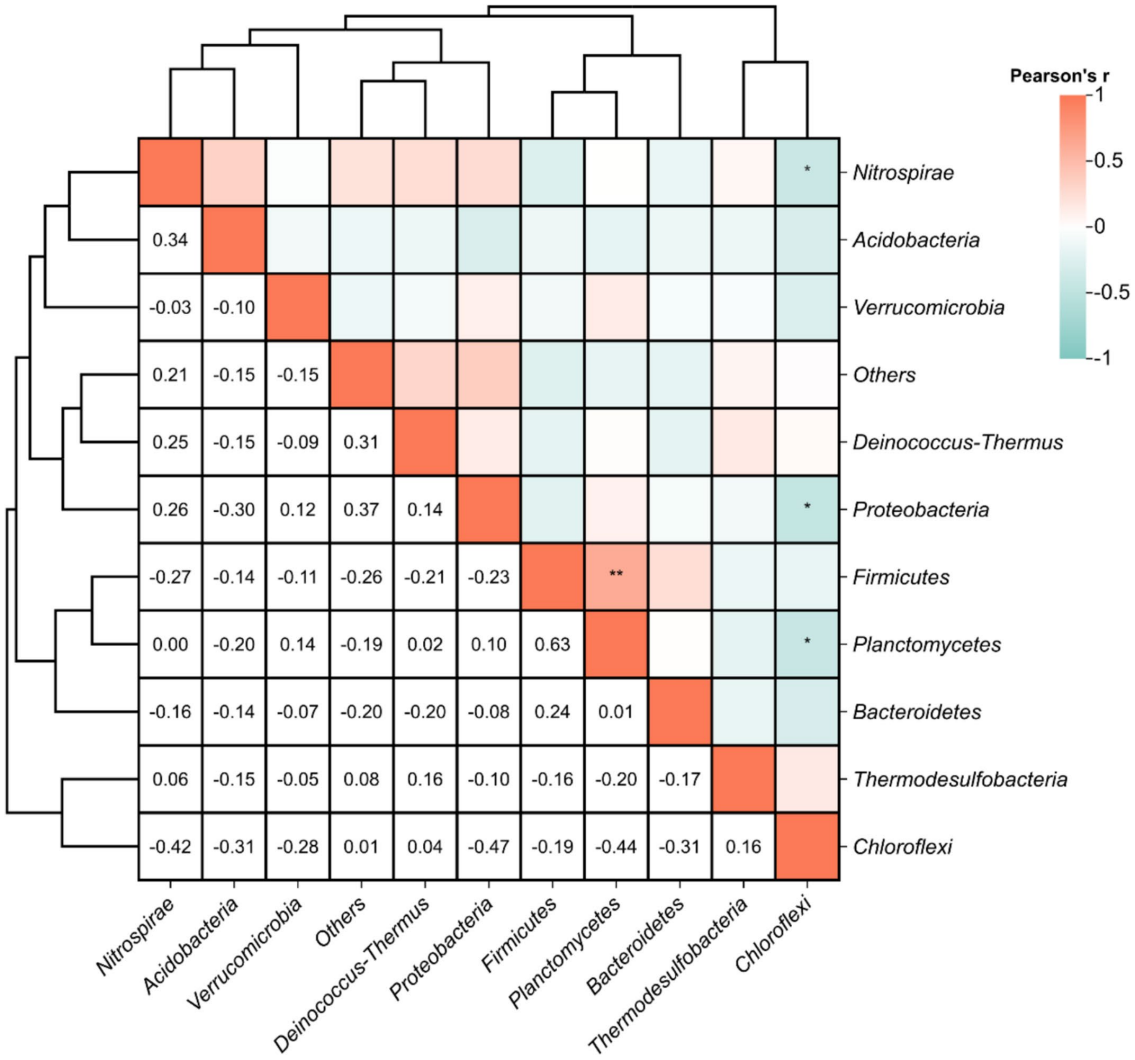
Pearson correlations between relative abundance of functional DNRA microbial populations. Statistical significance is marked as follows: *p ≤ 0.05, **p ≤ 0.01, ***p ≤ 0.001 and ****p ≤ 0.0001, and no * for no significant difference.

Pearson correlation analysis at the phylum level revealed significant interspecific interactions among DNRA microbial populations (Figure 7). A strong positive correlation was identified between *Firmicutes* and *Planctomycetes* (*r* = 0.63, *p* < 0.01), suggesting potential cooperative interactions in maintaining nitrogen retention functions within the hot spring ecosystems. Conversely, significant negative correlations were observed between *Chloroflexi* and *Planctomycetes* (*r* = −0.44), *Proteobacteria* (*r* = −0.47), and *Nitrospirae* (*r* = −0.42) (*p* < 0.05), potentially indicating niche competition among these taxa. These antagonistic relationships may reflect divergent metabolic strategies or resource partitioning in the geochemically heterogeneous thermal habitats.

### Correlation analysis of DNRA communities and physicochemical parameters

**Figure 8:**
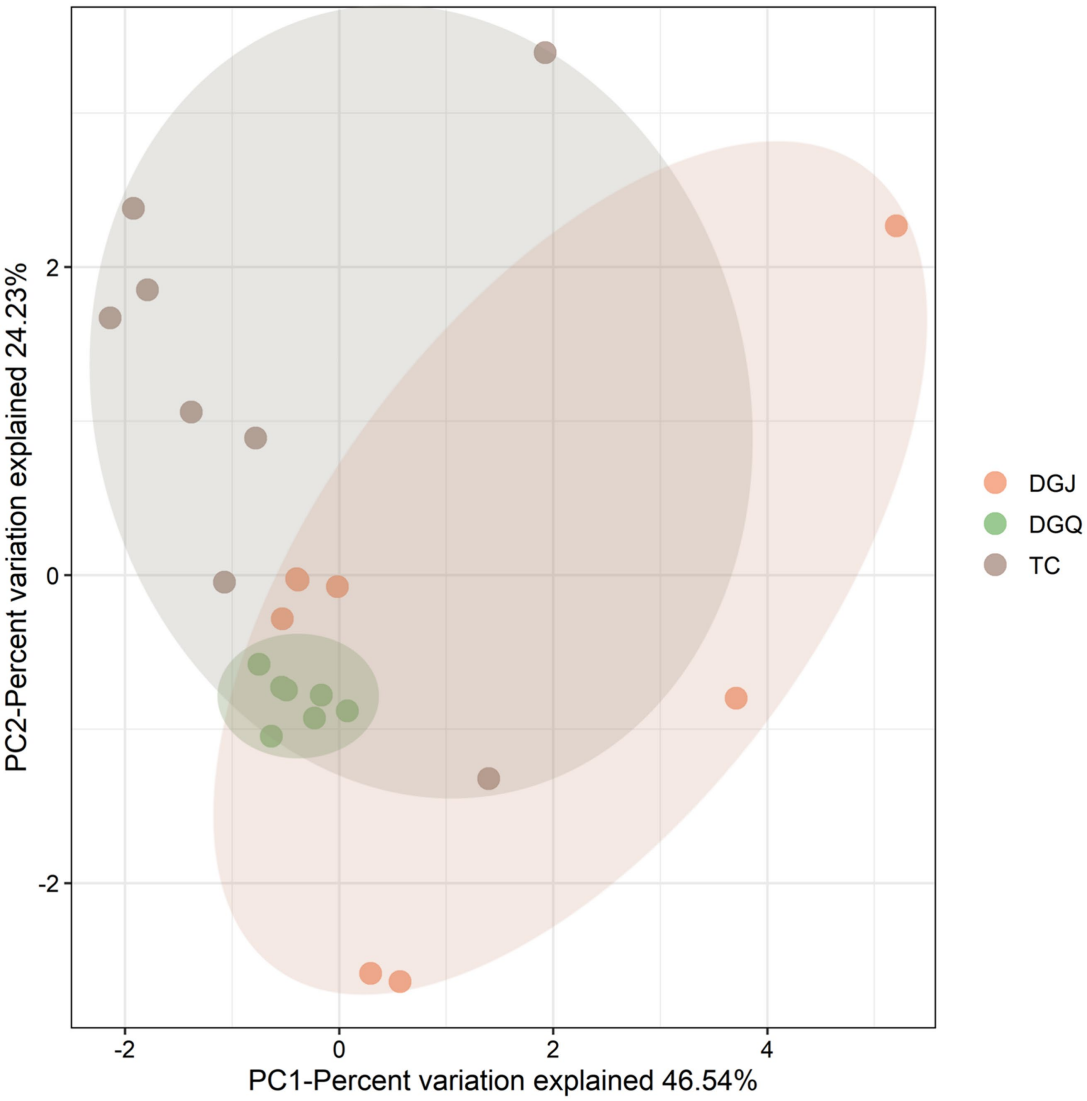
Principal component analysis (PCA) of positive sample sites across the three sampling regions (TC, DGJ, and DGQ), based on physicochemical parameters (pH, temperature, ion concentrations, etc.).

**Figure 9:**
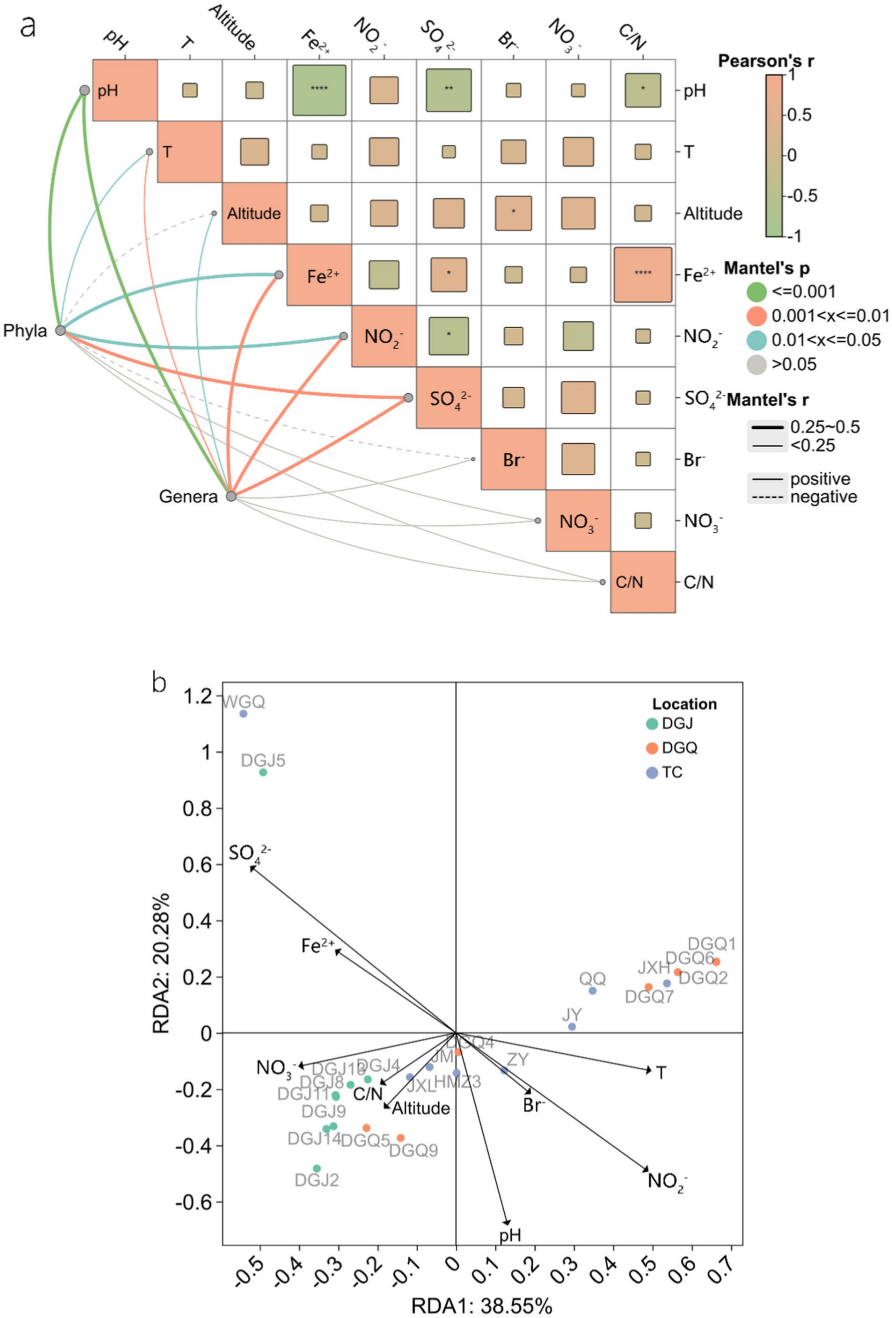
Mantel test of physicochemical factors at the phylum and genus level **(a)**, RDA analysis of DNRA microbial communities in relation to physicochemical factors **(b)**, the arrow length represents the explanatory strength of environmental factors on DNRA community variation, with longer arrows indicating stronger influences. The angle between arrows and axes reflects their correlation with the principal components (smaller angles indicate higher correlation), while the perpendicular distance from sample points to arrows quantifies the impact of each factor on specific samples (closer distances imply stronger effects).

Principal component analysis (PCA) revealed significant differences in physicochemical properties among sampling sites, with the clearest differences observed between DGJ and TC, while DGQ sites had more consistent properties. The first principal component (PC1) accounted for 46.54% of the total environmental variation (Figure 8). Correlation analysis (Figure 9a) revealed several important relationships between physicochemical parameters and the *nrfA* gene abundance. Notably, pH was positively associated with DNRA microbial community diversity. Redundancy analysis (RDA) confirmed that pH, SO₄^2−^ and NO₂^−^ concentrations significantly influenced the DNRA community composition and structure at most sampling sites (Figure 9b). Other factors such as NO₃^−^, C/N ratios and altitude also played different roles in shaping DNRA diversity. For example, sites such as DGJ5 and WGQ, which are characterized by high abundance of *Acidobacteria*, were strongly influenced by SO₄^2−^ concentrations.

**Figure 10:**
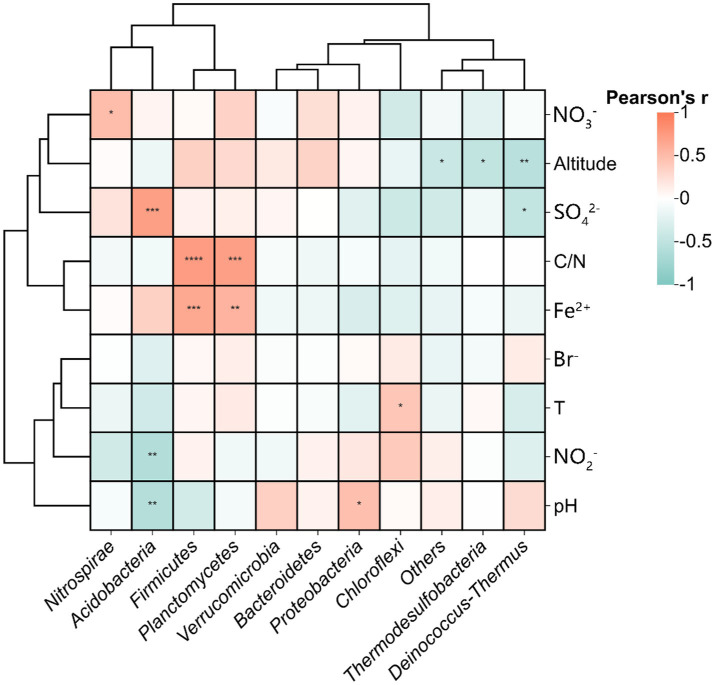
Pearson correlations between measured environmental variables and relative abundance of functional microbial populations in DNRA, Statistical significance is marked as follows: *p ≤ 0.05, **p ≤ 0.01, ***p ≤ 0.001 and ****p ≤ 0.0001, and no * for no significant difference.

Environmental drivers of phylum distributions were further quantified through Pearson correlations (Figure 10). *Thermodesulfobacteria* and *Deinococcus-Thermus* showed negative correlations with elevation, suggesting suppressed activity in high-altitude, low-pressure environments. *Proteobacteria* and *Firmicutes* were positively linked to Fe^2+^concentrations, aligning with their known roles in iron redox cycling. *Acidobacteria* displayed dual negative correlations with pH and NO_2_^−^ but a strong positive association with SO_4_^2−^, underscoring their adaptation to sulfidic, acidic niches. Notably, *Chloroflexi* and *Nitrospirae* showed no significant temperature dependence, highlighting DNRA functional resilience to thermal fluctuations.

**Figure 11:**
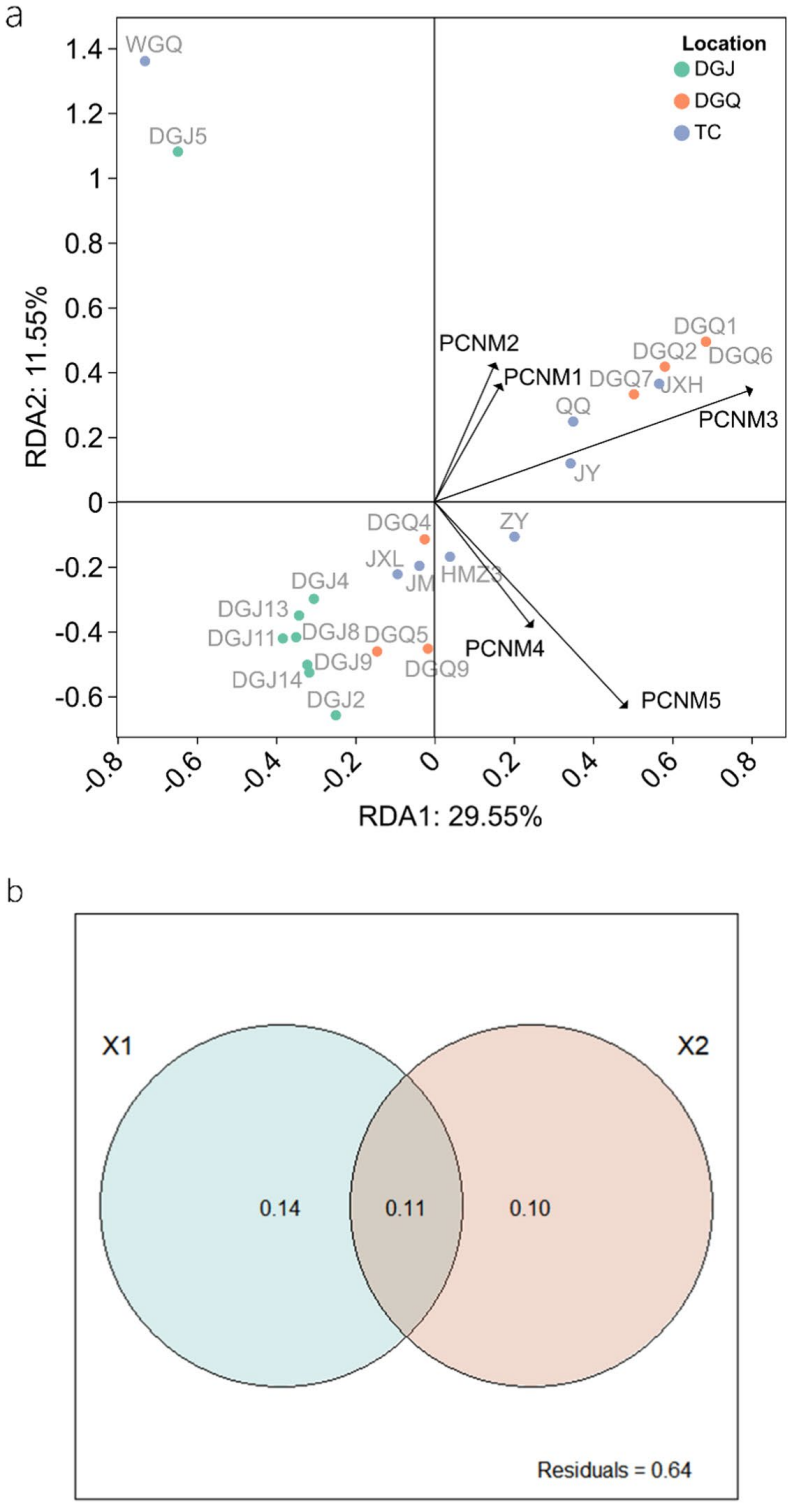
RDA analysis illustrating the influence of spatial factors and DNRA microbial community structure **(A)**, Variance Partitioning Analysis (VPA) quantifying the relative contributions of physicochemical (X1) and spatial (X2) factors on DNRA microbial community structure **(B)**.

Spatial factors also contributed significantly to DNRA community structure. Principal coordinates of neighbor matrices (PCNM) analysis identified PCNM3 as a critical vector influencing microbial composition across sites (Figure 11a). Variance partitioning analysis (VPA) revealed that physicochemical and spatial factors together explained about 36% of the variation in DNRA microbial community structure, while 64% remained unexplained (Figure 11b). Of the explained variation, physicochemical and spatial factors and their combined effect accounted for 14, 10 and 11%, respectively. It is important to note that correlations between microbial taxa abundances, while suggestive of potential interactions, do not confirm direct ecological relationships. These patterns may arise from shared environmental preferences, unmeasured variables, or stochastic processes (Carr et al., 2019). Future experimental validations are required to infer causality.

## Discussion

In this study, we investigated the microbial communities that mediate dissimilatory nitrate reduction to ammonium (DNRA) processes in sediment samples collected from three hot spring regions in Yunnan and Tibet. Our aim was to understand the distribution, community structure, and diversity of microorganisms harboring *nrfA* genes, and to investigate the environmental and spatial factors that influence their composition.

### DNRA microbial diversity in the Tibet-Yunnan hot springs

The DNRA microbial communities in the hot springs of Yunnan and Tibet were predominantly composed of the phyla *Chloroflexi* and *Proteobacteria*, which are also dominant in aquatic environments worldwide (e.g., wetland and other sediment-rich aquatic ecosystems) (Mark et al., 2016). The high abundance of *Thermoflex* is in hot springs indicates their potential role in the sulfur coupled DNRA pathway, a mechanism previously associated with nitrogen conservation in sulfide systems. Interestingly, the thermophilic *Deinococcus-Thermus* group was also found to harbor the *nrfA* functional gene, suggesting the presence of microorganisms harboring *nrfA* genes endemic to high-temperature environments. Among these thermophiles, *Thermus* species is one of the most widespread genera, with isolates occurring in both natural and anthropogenic thermal environments. Its high growth rate, high yield of cultured cells, and constitutive expression of efficiently activated organs make this strain an excellent laboratory model for studying the molecular basis of thermophilic bacteria (Cava et al., 2009), its metabolic pathways cover multiple modes such as aerobic respiration and incomplete denitrification. This process, mediated by enzymes such as nitrate and nitrite reductases, allows *Thermus* to reduce nitrate to nitrous oxide (N_2_O) or nitric oxide (NO), contributing to nitrogen flux in thermal ecosystems (Mefferd et al., 2022; Murugapiran et al., 2013). While our study identifies microorganisms carrying *nrfA* genes, functional validation (e.g., metatranscriptomics or dynamic experiment) is required to confirm active DNRA. This finding provides a valuable basis for the isolation and study of thermophilic DNRA microorganisms as experimental models. The DNRA microbial populations in these hot springs were different from those in other high-temperature environments, such as *Verrucomicrobia* (Ward et al., 2019), *Thermovibrio ammonificans* (Vetriani et al., 2004), *Caldithrix abyssi* (Miroshnichenko et al., 2003), *Candidatus Thermoflexus japonica* (Kato et al., 2018), *Ammonifex degensii* (Hafenbradl et al., 1996), and *Chloroflexus* (Bennett et al., 2020). However, several of the top ten phyla recovered in this study were isolated from other environments in previous studies. For example, Welsh et al. (2014) identified six phyla containing the *nrfA* gene by sequencing, namely *Chloroflexi*, *Proteobacteria*, *Planctomycetes*, *Acidobacteria*, *Verrucomicrobia* and *Firmicutes* (Welsh et al., 2014). Correlation analysis revealed significant positive interactions among certain populations, such as *Firmicutes* and *Planctomycetes*, which may explain similarities in the microbial compositions of DNRA in different habitats. This suggests that DNRA community structures are shaped by complex ecological and environmental interactions. Future work should prioritize coupling gene surveys with activity measurements. Specifically, validating DNRA functionality in hot springs is our next priority. We propose to validate the DNRA function of hot spring microorganisms using pure culture microorganisms containing the *nrfA* gene in conjunction with transcriptomics and through microcosmic simulation experiments.

### Influence of physicochemical factors on DNRA communities

Redundancy analysis (RDA) shows that nitrate (NO_3_^−^), pH and temperature (T) were the factors most strongly correlated with DNRA microbial community composition. However, the presence of the *nrfA* gene over a wide temperature range (40–80°C) suggests that temperature may not have a direct influence on the distribution of the DNRA microorganism, but rather on the community composition. Among these factors, pH was found to be a crucial determinant of *nrfA* gene distribution, consistent with previous studies showing that DNRA pathways generally function in wetland ecosystems at pH values above 6.0. These results suggest that the DNRA microbial community is shaped by unique combinations of environmental factors in different regions, highlighting the need for targeted isolation studies to understand specific population-environment interactions. Although dissimilatory nitrate reduction to ammonium (DNRA) has traditionally been linked to environments with high organic carbon availability (Liu et al., 2021), our findings in low-organic hot spring ecosystems suggest that sulfides (SO_4_^2−^/S^2−^) may serve as an alternative electron donor. This observation aligns with recent advancements in understanding sulfur cycling in heterotrophic microorganisms and DNRA processes, where sulfur-metabolizing microorganisms, particularly *Thiobacillus* species, have been identified to encode the *nrfA* gene (Li X. et al., 2022). Notably, elemental sulfur has been demonstrated to stimulate DNRA activity (Li S. et al., 2022). Specifically, the sulfide oxidation coupled with DNRA (Sox-DNRA pathway) has emerged as a crucial nitrogen retention mechanism in nutrient-depleted thermal environments (Wang et al., 2021), highlighting the adaptability of microbial nitrogen cycling strategies in extreme ecosystems. The threshold effects of key environmental parameters exhibited marked regional specificity. Our redundancy analysis (RDA) revealed distinct drivers across regions: DGJ communities were co-regulated by altitude, NO_3_^−^, and C/N ratios, whereas TC communities were predominantly influenced by pH and Fe^2+^ concentrations (Figure 9b). This spatial heterogeneity likely stems from contrasting geological settings. In DGJ, the extreme altitude (>5,000 m) reduces atmospheric pressure, potentially enhancing upward migration of reduced subsurface fluids (Zhang et al., 2022). Conversely, the granitic bedrock in TC provides abundant Fe^2+^, possibly supporting iron oxidation-coupled DNRA pathways (Bai et al., 2023). The pivotal role of pH aligns with prior studies showing DNRA activation thresholds above pH 6.0 in wetlands (Burgin and Hamilton, 2007), suggesting alkaline conditions may optimize energy acquisition through enhanced proton motive force. Notably, our observed pH-*nrfA* correlation extends this paradigm to high-temperature ecosystems, underscoring pH as a universal regulator across biomes.

### Influence of spatial factors on DNRA communities

Spatial heterogeneity analysis demonstrated significant structural divergence among regions. While DGQ and TC exhibited relatively homogeneous communities, DGJ displayed higher variability—likely attributable to intermittent hydrothermal activity and complex vent distributions (Guo et al., 2020). Contrasting geological frameworks further explain these patterns: DGQ’s Quaternary carbonate strata create stable thermal environments (Zhang H.-s. et al., 2023), whereas TC’s fractured granite cover (Guo et al., 2014) permits dynamic fluid exchange. Principal Component Analysis (PCA) of physicochemical parameters (Figure 8) corroborated this environmental dichotomy, with DGJ-TC divergence reflecting differential deep fluid migration pathways and meteoric water mixing ratios shaped by Tibetan Plateau uplift. However, VPA indicated that 64% of community variation remained unexplained by measured factors. Three plausible mechanisms may account for this residual variance: (1) microbial interaction networks, such as the negative correlations between Chloroflexi and Acidobacteria (Figure 7); (2) trace metals (e.g., Mo, Cu) could modulate NrfA enzyme activity, as demonstrated in marine DNRA communities (Wetherington et al., 2022); (3) historical dispersal limitations may drive stochastic priority effects, where early colonizers preempt ecological niches. These findings underscore the multiscale nature of DNRA community assembly in extreme environments, governed by intertwined environmental filtering, biotic interactions, and stochastic processes. While this study provides a foundational characterization of microbial communities in geothermal springs, future investigations should adopt hypothesis-driven approaches to unravel specific functional mechanisms, such as the role of DRNA pathways in extremophile adaptation. Integrating multi-omics data with controlled environmental simulations could validate these hypotheses and bridge descriptive patterns with causal relationships.

## Conclusion

This study reveals the widespread distribution of DNRA microorganisms in Tibet-Yunnan hot springs, with *nrfA* genes detected in 23 sediment samples spanning 38–80°C. Dominant populations included *Chloroflexi* (34.2%) and *Proteobacteria* (24.3%), while the thermophilic *Deinococcus-Thermus* (1.1%) demonstrated DNRA potential at extreme temperatures. Genus-level resolution identified *Thermoflexus (Chloroflexi)* as a key taxon in sulfur-rich springs, likely coupling sulfur oxidation to DNRA, and *Geothrix (Acidobacteria)* in acidic sites, suggesting niche-specific adaptations for nitrate reduction. The key environmental factors influencing DNRA microbial community composition include pH, sulfate (SO₄^2−^), and nitrite (NO₂^−^) concentrations. They were found to be the key factors affecting the distribution and characteristics of DNRA microorganisms in hot springs. These findings position DNRA as a critical ammonium source in geothermal ecosystems, particularly where sulfide-rich, organic-poor conditions limit nitrogen fixation. The prevalence of sulfur-linked DNRA taxa (*Thermoflexus, Thiobacillus*) aligns with recent evidence of sulfur-driven nitrogen conservation in thermal niches. Furthermore, the thermal resilience of *Deinococcus-Thermus* supports hypotheses about nitrogen cycle functionality on early Earth, where analogous environments may have sustained primordial life. Future work should prioritize isolating these thermophiles to validate enzymatic activity and explore their evolutionary adaptations to extreme biogeochemical regimes.

## Data Availability

The original contributions presented in the study are publicly available. This data can be found: https://www.ncbi.nlm.nih.gov/, accession number: PRJNA1193295.

## Footnotes

^1^ https://www.arb-silva.de/

^2^ https://ftp.ncbi.nlm.nih.gov/blast/db/FASTA/

^3^ https://www.chiplot.online
