# Supplementary material for: The nrfA-type microbial communities are widespread in hot springs of the Tibet-Yunnan geothermal zone
Source: Front Microbiol. 2025 Jul 15;16:1540611. doi: 10.3389/fmicb.2025.1540611 (PMC12303954; doi:10.3389/fmicb.2025.1540611)
Supplement: Supplementary file 1 [file Supplementary_file_1.docx]

**The *nrfA*-type microbial communities are widespread in hot springs of the Tibet-Yunnan geothermal zone**

**Xi Chen, Geng Wu^*^, Zhicheng Yu, Fangmin Li, Hongchen Jiang**

^1^State Key Laboratory of Biogeology and Environmental Geology, China University of Geosciences, Wuhan, China

*** Correspondence:**Corresponding Geng Wu
Geng Wu, wugeng@cug.edu.cn

**Section S1: Statistical testing methods used in diversity analysis**

In this study, we used the R language vegan package to analyze community ecology, including alpha diversity and beta diversity (PCoA) of the community. In beta analysis, use the default Bray Curtis distance of vegan's *vegdist* function (applicable to non-parametric dissimilarity of species abundance data). And use the *pcoa* function of APE to reduce the dimensionality of the distance matrix, extract the first two principal coordinates for visualization, and perform principal coordinate analysis (PCoA). In alpha analysis, the OTU table was diluted to the minimum sample sequencing depth using vegan's permutation to reduce the impact of sequencing volume differences on diversity calculations. And calculate the Shannon Wiener index, Simpson index, and Observed OTUs (richness). After the calculation is completed, we use Shapiro Wilk to test for normality. The Shannon Wiener index and Observed OTUs (richness) data follow a normal distribution, so we use ANOVA for testing and Bartlett's test for homogeneity of variance; The Simpson index does not follow a normal distribution, therefore Kruskal Wallis test is used, followed by Dunn test and Bonferroni correction.

**Section 2: Materials and Methods**

(1) Study Area and Sampling Context

The research was conducted in high-altitude environments (elevation >3,000 m) across three geologically distinct regions: Tengchong (TC) in Yunnan Province, and Dagejia (DGJ) and Duoguoqu (DGQ) in Tibet. These areas exhibit extreme climatic conditions, including intense solar radiation, low annual temperatures (mean <5°C), pronounced diurnal temperature fluctuations (>15°C), and seasonal drought. Due to severe winter conditions (mean temperature <-10°C) and heavy snowfall, field sampling was exclusively conducted during the spring and summer months (April–August 2023), coinciding with active hydrothermal venting and partial thawing of permafrost. This seasonal window ensured safe access to thermal springs and enabled the collection of representative microbial-geochemical samples.

(2) Field Sampling Procedures

Water and Sediment Collection: At each hydrothermal site, triplicate water and sediment samples were collected for molecular, cultivation, and geochemical analyses. Water samples were obtained directly from spring vents using pre-sterilized 50 ml polypropylene bottles. For molecular studies, 40 ml of water was immediately filtered through 0.22 μm cellulose acetate membranes (Sartorius) and stored in dry ice. Unfiltered water aliquots for cultivation experiments were preserved at 4°C. Sediment cores (0–10 cm depth) were extracted using ethanol-sterilized stainless steel scoops, transferred to sterile 15 ml centrifuge tubes (Corning), and flash-frozen in dry ice.

Specialized protocols were implemented for dissolved organic carbon (DOC) and dissolved inorganic carbon (DIC) sampling to minimize contamination. DOC samples were collected after inorganic parameter measurements using syringe filtration systems equipped with pre-combusted GF/F membranes (Whatman). After pre-rinsing with 20-30 ml of spring water, 20 ml of filtrate was collected in acid-washed glass vials, ensuring no contact between the filter outlet and vial surfaces. Field blanks (Milli-Q water processed identically) were included for quality control. DIC samples were collected in 40 ml serum bottles triple-rinsed with source water, filled via submerged tubing to prevent gas exchange, and preserved with 50 μl saturated HgCl_2_ before refrigeration at 4°C.

(3) In Situ Geochemical Characterization

On-site measurements were performed using calibrated field instruments. Geographic coordinates were recorded using a Garmin eTrex H GPS unit in differential mode. Water temperature and pH were measured in triplicate with a portable SX711 pH meter (Sanxin, China), calibrated daily with pH 4.0, 7.0, and 10.0 buffers. Nitrogen species (NO_2_^-^) and ferrous iron (Fe^2+^) concentrations were quantified using Hach DR300 spectrophotometer with dedicated reagent kits (Fe^2+^, reagent number 103769; NO_2_^-^, reagent number 2107569).

(4) Laboratory Processing and Analytical Workflows

Ionic and Trace Element Analysis: Major cations (Na^+^, K^+^, Ca^2+^, Mg^2+^) and anions (Br^-^, NO_3_^-^, SO_4_^2-^) were analyzed using a Dionex DX-600 ion chromatograph (Thermo Fisher Scientific). Cations were separated on a CS12A column with 20 mM methanesulfonic acid eluent, while anions were resolved on an AS11-HC column under a KOH gradient. Trace elements were quantified via inductively coupled plasma mass spectrometry (Agilent 7700 ICP-MS) in helium collision mode, calibrated against NIST 1640a reference materials.

Organic Carbon Quantification: Total organic carbon (TOC) and dissolved organic carbon (DOC) were determined using a NC 2100S analyzer (Analytik Jena). Samples were acidified with 2M HCl to remove inorganic carbon prior to high-temperature combustion (850°C) and non-dispersive infrared detection. Method detection limits were 0.1 mg C/L, validated through daily analysis of Deep Atlantic seawater reference material.

(5) Quality Assurance and Control

Triplicate sampling at each site ensured statistical robustness. Field blanks and certified reference materials (SLRS-6 for cations, NASS-7 for anions) were processed with each analytical batch. All instruments maintained relative standard deviations (RSD) below 5% for ion chromatography, 10% for ICP-MS, and 3% for TOC analyses. Sampling metadata, including weather conditions, spring discharge rates, and visible microbial mat characteristics, were systematically documented in field logs.

Supplementary Table S1: Sequencing depth and coverage metrics for *nrfA* gene libraries across 23 samples

| Samples | Amount of sequencing data | Amount of sample data off machine | barcode reads | Ratio (%) |
| --- | --- | --- | --- | --- |
| DGJ2 | 6W raw reads | 187615 | 198104 | 94.71 |
| DGJ4 | 6W raw reads | 99267 | 103570 | 95.85 |
| DGJ5 | 6W raw reads | 120441 | 125726 | 95.8 |
| DGJ8 | 6W raw reads | 285858 | 303360 | 94.23 |
| DGJ9 | 6W raw reads | 178579 | 186473 | 95.77 |
| DGJ11 | 6W raw reads | 247321 | 259024 | 95.48 |
| DGJ13 | 6W raw reads | 342247 | 360642 | 94.9 |
| DGJ14 | 6W raw reads | 113580 | 119360 | 95.16 |
| DGQ1 | 6W raw reads | 192400 | 201445 | 95.51 |
| DGQ2 | 6W raw reads | 202422 | 213154 | 94.97 |
| DGQ4 | 6W raw reads | 203054 | 212843 | 95.4 |
| DGQ5 | 6W raw reads | 195985 | 204440 | 95.86 |
| DGQ6 | 6W raw reads | 179047 | 187117 | 95.69 |
| DGQ7 | 6W raw reads | 210628 | 219452 | 95.98 |
| DGQ9 | 6W raw reads | 89917 | 93587 | 96.08 |
| HMZ3 | 6W raw reads | 241861 | 256495 | 94.29 |
| JXH | 6W raw reads | 181346 | 191616 | 94.64 |
| JXL | 6W raw reads | 204762 | 214168 | 95.61 |
| JM | 6W raw reads | 131871 | 138060 | 95.52 |
| JY | 6W raw reads | 113818 | 118536 | 96.02 |
| ZY | 6W raw reads | 74891 | 77768 | 96.3 |
| WGQ | 6W raw reads | 151203 | 158383 | 95.47 |
| QQ | 6W raw reads | 342197 | 359796 | 95.11 |

Supplementary Table S2: Alpha diversity indices (Shannon, Simpson, Observed OTUs) for DNRA microbial communities across sampling regions

| Location | Sample | Total_reads | Observed_OTUs | Shannon_Wiener | Simpson |
| --- | --- | --- | --- | --- | --- |
| DGJ | DGJ2 | 170170 | 356 | 3.109 | 0.889 |
|  | DGJ4 | 91791 | 60 | 1.342 | 0.626 |
|  | DGJ5 | 112429 | 92 | 0.841 | 0.358 |
|  | DGJ8 | 266210 | 190 | 2.436 | 0.774 |
|  | DGJ9 | 163413 | 141 | 2.637 | 0.888 |
|  | DGJ11 | 228990 | 197 | 1.289 | 0.433 |
|  | DGJ13 | 296012 | 254 | 2.416 | 0.807 |
|  | DGJ14 | 103177 | 94 | 2.053 | 0.829 |
| DGQ | DGQ1 | 178480 | 255 | 1.481 | 0.376 |
|  | DGQ2 | 183555 | 377 | 2.066 | 0.541 |
|  | DGQ4 | 187336 | 301 | 2.834 | 0.858 |
|  | DGQ5 | 177357 | 267 | 2.494 | 0.738 |
|  | DGQ6 | 165263 | 373 | 1.806 | 0.467 |
|  | DGQ7 | 193676 | 408 | 2.662 | 0.767 |
|  | DGQ9 | 84538 | 314 | 1.477 | 0.488 |
| TC | HMZ3 | 213819 | 402 | 3.571 | 0.903 |
|  | JXH | 165937 | 147 | 1.786 | 0.632 |
|  | JXL | 164649 | 264 | 3.364 | 0.936 |
|  | JM | 110003 | 153 | 2.896 | 0.899 |
|  | JY | 105899 | 158 | 2.973 | 0.918 |
|  | ZY | 69603 | 140 | 2.786 | 0.877 |
|  | WGQ | 141995 | 177 | 1.148 | 0.457 |
|  | QQ | 310352 | 309 | 2.255 | 0.795 |


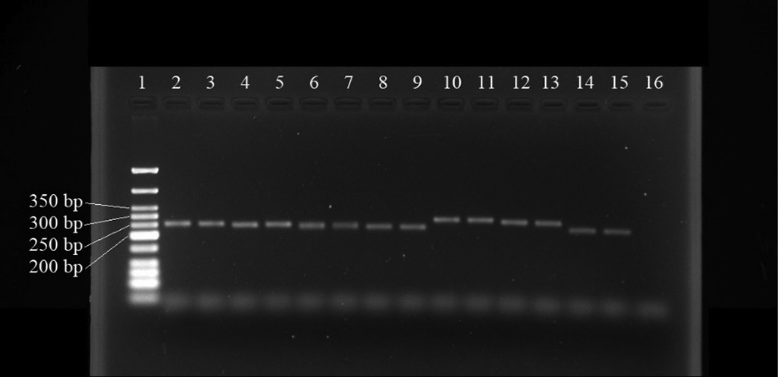


**Supplementary Figure S1:** Gel electrophoresis of *nrfA* primer validation from Cannon et al. (2019). Gel electrophoresis image of PCR products amplified from DNA of seven reference organisms using primer pair nrfAF2awMOD / nrfAR1MOD. Lane 1: DNA ladder, Lanes 2–3: Bacillus UAAc-7, Lanes 4–5: S. oneidensis MR-1, Lanes 6–7: A. dehalogenans 2CP-1, Lanes 8–9: D. hafniense DCB-2, Lanes 10–11: D. vulgaris str. Hildenborough, Lanes 12–13: W. succinogenes, Lanes 14–15: S. fonticola HAc-5, and Lane 16: ultrapure water.


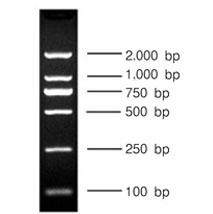


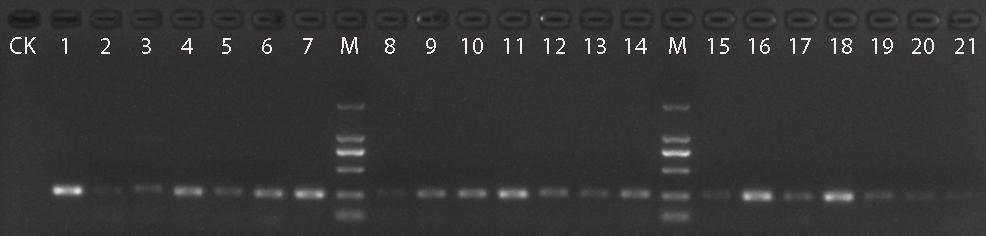

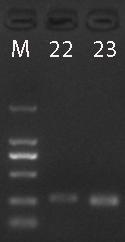


**Supplementary Figure S2:** Electropherograms of *nrfA* gene PCR amplification from 23 hot spring sediment samples. Agarose gel electrophoresis (1.5%) of nrfA PCR products from 23 sediment samples. Lanes 1-23: Amplified nrfA fragments (~270 bp); Lane M: DNA ladder (100-1,000 bp).


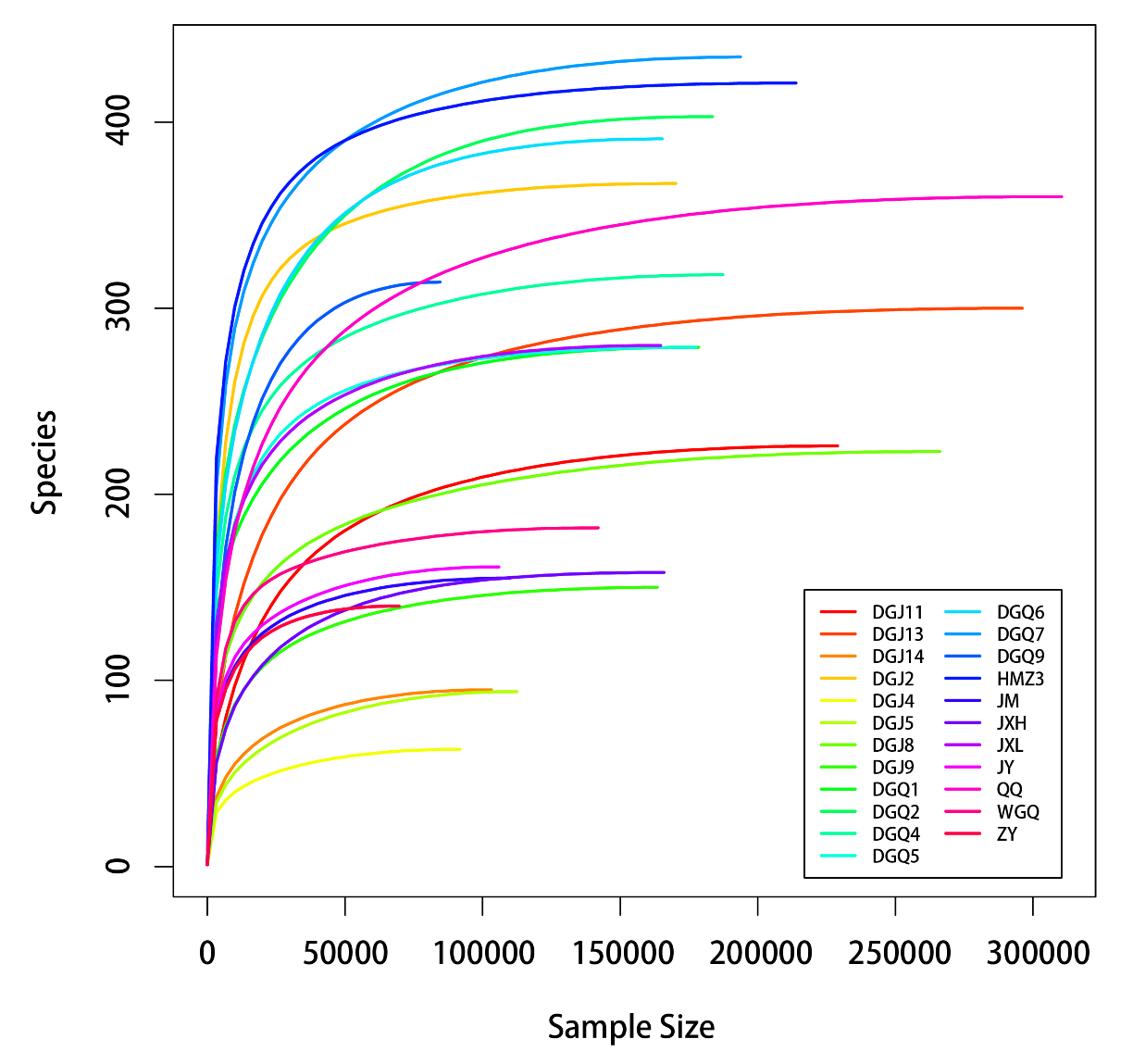


**Supplementary Figure S3:** Rarefaction curves of *nrfA* gene sequencing across 23 hot spring samples
